# Chat- and internet-based cognitive–behavioural therapy in treatment of adolescent depression: randomised controlled trial

**DOI:** 10.1192/bjo.2018.18

**Published:** 2018-06-26

**Authors:** Naira Topooco, Matilda Berg, Sofie Johansson, Lina Liljethörn, Ella Radvogin, George Vlaescu, Lise Bergman Nordgren, Maria Zetterqvist, Gerhard Andersson

**Affiliations:** Department of Behavioural Sciences and Learning, Linköping University, Linköping, Sweden; Department of Behavioural Sciences and Learning, Linköping University, Linköping, Sweden, and Department of Clinical Neuroscience, Karolinska Institutet, Stockholm, Sweden; Centre for Social and Affective Neuroscience, Department of Clinical and Experimental Medicine and Child and Adolescent Psychiatry, Linköping University, Linköping, Sweden; Department of Behavioural Sciences and Learning, Linköping University, Linköping, Sweden, and Department of Clinical Neuroscience, Karolinska Institutet, Stockholm, Sweden

**Keywords:** Cognitive–behavioural therapy, blended treatment, adolescent, depression, treatment gap, stigma, internet-based treatment, internet-supported, digital, iCBT

## Abstract

**Background:**

Depression is a major contributor to the burden of disease in the adolescent population. Internet-based interventions can increase access to treatment.

**Aims:**

To evaluate the efficacy of internet-based cognitive–behavioural therapy (iCBT), including therapist chat communication, in treatment of adolescent depression.

**Method:**

Seventy adolescents, 15–19 years of age and presenting with depressive symptoms, were randomised to iCBT or attention control. The primary outcome was the Beck Depression Inventory II (BDI-II).

**Results:**

Significant reductions in depressive symptoms were found, favouring iCBT over the control condition (F(1,67) = 6.18, *P* < 0.05). The between-group effect size was Cohen's *d* = 0.71 (95% CI 0.22–1.19). A significantly higher proportion of iCBT participants (42.4%) than controls (13.5%) showed a 50% decrease in BDI-II score post-treatment (*P* < 0.01). The improvement for the iCBT group was maintained at 6 months.

**Conclusions:**

The intervention appears to effectively reduce symptoms of depression in adolescents and may be helpful in overcoming barriers to care among young people.

**Declaration of interest:**

N.T. and G.A. designed the programme. N.T. authored the treatment material. The web platform used for treatment is owned by Linköping University and run on a non-for-profit basis. None of the authors receives any income from the programme.

Unipolar depressive disorders are a major cause of disability-adjusted life years among adolescents globally.^1^ It is estimated that only 20% of young people with mental healthcare needs receive treatment, owing to the limited treatment resources and to individual barriers of social stigma and poor mental health literacy.^2^ Technology-assisted treatments are of great importance in enhancing mental healthcare capacity. There is ample research supporting the effects of online interventions based on cognitive–behavioural therapy (iCBT) for common mental disorders in adult populations.^3^ iCBT prevention and treatment programmes targeting youth depression are promising,^2^^,^^4^ but studies are limited in number and heterogeneous in population. iCBT is expected to fit adolescents particularly well, because they are avid internet users; however, the population presents challenges in terms of acceptability of treatment, for example, reflected in attitudes towards programmes,^5^^–^^7^ and in reported recruitment^6^^,^^8^ and completion rates.^9^^,^^10^ A general consideration is that iCBT programmes are often designed for self-help only or with restricted asynchronous support (≤15 min weekly) to address treatment shortages.^3^ It could be questioned whether this is the optimal approach for young people. Adolescents may benefit from and need more immediate feedback, and interventions with no real-time interaction may be insufficiently reinforcing. Young people's preference for real-time online communication, e.g. instant messaging (chat), is well documented.^11^ Indeed, early evidence on iCBT for young people suggests that programmes with no therapist contact are less promising than supported or internet and face-to-face blended interventions,^12^^–^^15^ which is line with findings for adult populations.^16^ Young individuals are at an early stage in the disorder, and we know that untreated depression often follows them into adulthood and predicts a range of negative outcomes in psychiatric and physical health.^17^ More intense internet interventions that focus on effectively averting a prolonged course of illness can be of significant value to the individual and society.^18^^,^^19^

## A text-based blended treatment approach

Novel ‘blended’ treatment approaches that combine therapist sessions (face-to-face, or via video, telephone or chat) with online self-help components have emerged as a strategy to maintain an active therapist–patient collaboration in treatment, while providing reliable support in the form of online self-help components to reduce therapist time in comparison with standard treatment.^16^^,^^20^^,^^21^ A blended approach and, moreover, the use of chat communication instead of traditional face-to-face meetings may be particularly beneficial for young individuals. Discussing sensitive subjects and sharing and reflecting on feelings and thoughts in a safe environment (e.g. home) may facilitate autonomy and reduce stigma.^7^^,^^22^ Benefits associated with written communication and elimination of the pressure of sitting opposite a physical person have previously been reported for chat-based helplines for youth.^22^ In terms of treatment access and cost, therapist time may potentially be reduced in comparison with standard treatment delivery. Concurrent chat sessions are considered a key benefit of chat,^23^ and the feasibility of multiple sessions in counselling has been established with chat helplines.^22^ The basic and available technology is an advantage, as the development of iCBT programmes for young people has tended to be slow and/or costly.^24^ Chat-based psychological support is a growing area in mental healthcare;^25^ however, to date, only one controlled study has evaluated chat sessions in CBT depression treatment for adolescents using a group format.^14^ The current study contributes to the area, by the development and evaluation of an iCBT programme that includes therapist sessions using chat communication. The main objective was to evaluate the effects of the iCBT programme on adolescent depression compared with an attention control condition. We hypothesised that real-time therapist contact would improve outcome and treatment engagement compared with self-help or less-supported iCBT programmes. The secondary aim of the study was to explore aspects of target audience acceptability. To investigate whether the treatment approach would be attractive to young individuals in need of mental healthcare assistance, participants were recruited from the community by means of self-selection.

## Method

### Trial design

Adolescents 15–19 years of age suffering from depressive symptoms were randomised at a 1 : 1 ratio to iCBT (*n* = 33) or an attention control condition (*n* = 37). Outcome variables were defined *a priori* and assessments were made at baseline, post-treatment (8 weeks) and at 6 months. Recruitment and assessment took place from January to February, treatments were delivered from February to April, and follow-up assessments were completed in October 2015. Participants in the control condition were offered treatment after the post-treatment assessment and were not included in follow-up analyses. All participants provided informed consent. The study recommended participants to inform guardians of their participation, but this was not mandatory. The study included investigation of potential negative effects from treatment.^26^ Ethics approval was granted by the regional ethics committee in Östergötland (reg. no. 2014/427-31). The study was registered at ClinicalTrials.gov (NCT02363205).

### Participants

Eligibility criteria for participants to take part in the present study were: 15–19 years of age and deemed to have sufficient maturity to participate in research; scoring 14 or more on the Beck Depression Inventory II (BDI-II); presenting with at least five symptoms of or fulfilling diagnosis of major depressive disorder according to the Mini-International Neuropsychiatric Interview (MINI) version 6.0;^27^ no severe suicidal ideation according to section B of the MINI (cut-off ≤16) or the suicidal ideation item (cut-off ≤1) in the Patient Health Questionnaire 9 (PHQ-9);^28^ no severe comorbid psychiatric condition that might interfere with the treatment (e.g. bipolar disorder or schizophrenia), assessed using the MINI; not currently undergoing psychotherapy treatment; no other medical problems that would require other treatments; not currently fulfilling the diagnostic criteria for alcohol or substance misuse according to the MINI and the Alcohol Use Disorders Identification Test.^29^ Participants with comorbid anxiety disorders were accepted if depression was the primary concern. Those currently taking medication for attention-deficit hyperactivity disorder, anxiety or depression were accepted, if the dose had been fixed during the past month and was kept constant throughout the study.

### Recruitment and inclusion procedure

Participants were recruited from the community by means of self-selection. Information about the study was posted on social media sites, and distributed to approximately 50 secondary and upper-secondary schools, and among Swedish organisations for youth mental health. Potential participants were directed to a study website that introduced the research project and enabled online registration. The website presented: (a) eligibility criteria; (b) how the treatment was to be conducted; (c) what to expect in terms of workload, (d) events taking place subsequent to registry and inclusion; (e) the project group with study therapists; and (f) a frequently asked questions section, based on questions found on mental health internet forums for young people. In the first step, the prospective participant registered for the study by confirming their understanding of the conditions presented on the website and providing informed consent. Thereafter, an email was sent to the prospective participant with instructions to complete an online screening consisting of outcome measures (accessed via an embedded web link). Completed screenings were reviewed within days to determine initial eligibility. Individuals deemed eligible were invited to a diagnostic interview (MINI) over the phone with study therapists. In the interviews, therapists assessed the presence of major depressive episode and other DSM-IV psychiatric disorders. Prospective participants confirmed their identity by providing their full name, address and personal identity number (PIN) in the interview. Swedish PINs are openly available and are used by authorities for the purpose of identification. The principal investigator reviewed all cases to determine final inclusion or exclusion. Eligible participants were randomised to either the treatment or the control condition, and the study therapist informed participants of the final decision over the telephone. Thereafter, treatments were initiated. Individuals who were excluded received a personal explanation and were offered guidance in seeking other healthcare services (which are free for young people in Sweden).

### Randomisation procedure

Following baseline assessment and informed consent, eligible patients were randomised. An independent researcher, not involved in the study, conducted the randomisation procedure by means of a computerised random number service. Participants were randomised in a 1 : 1 ratio using a block size of two or more. The last few participants were randomised at a single level, and one participant withdrew following randomisation (iCBT), resulting in an unequal number of participants in the two groups. It was not possible for participants or study therapists to be blinded to the treatment allocation, owing to the nature of the interventions.

### Treatment conditions

The iCBT programme was highly structured and based on previous iCBT programmes evaluated for adult depression^30^ that corresponded to a face-to-face CBT protocol for adult depression. The treatment was delivered over 8 weeks and consisted of eight skill-based modules and eight weekly chat sessions. Modules targeted behavioural and cognitive factors documented to reduce symptoms of depression and anxiety. Techniques included psychoeducation, behavioural activation, cognitive restructuring, affect regulation, anxiety management, and relapse prevention. Modules comprised reading material corresponding to 6–10 book pages, educational videos, fictional patient stories, interactive tasks and homework. Text materials were scored ‘very easy to read, fiction, popular newspapers’ when analysed using a computer-calculated readability index. Table 1 presents an overview of the treatment modules and homework. Modules were complemented with individual therapist chat sessions that lasted approximately 30 min. Chat sessions were structured and referred to content and homework in the previous and current treatment modules. Sessions assisted with treatment rationale, adjusting the homework level, problem identification and collaborative empiricism, as well as guidance and motivation for change. All treatment material and communication took place on a secured treatment platform that used a double authentication procedure at login. The platform was open 24 h a day and accessible from anywhere. No application was needed. Responsive web design was used to optimise the viewing experience across computer, tablet and smartphone. The chat interface developed for the study was similar to those used in instant messaging apps and included standard emojis. Screenshots of the intervention are presented in supplementary Appendix A (available at http://dx.doi/org/10.1192/bjo.2018.18).

**Table 1:** Treatment overview

Week	Module	Assignment/exercise
1	Psychoeducation depression	Write history, set goals
2	Analysis of behaviour	Identify dysfunctional and functional schemas
3	Behavioural activation	Mood–activity diary
4	Behavioural activation	Mood–activity diary
5	Cognitive restructuring	Identify and challenge thoughts
6	Psychoeducation anxiety	Anxiety management techniques, graded exposure
7	Emotional recognition	Coping strategies, self-esteem, affect regulation
8	Maintenance	Relapse prevention, treatment summary

The attention control consisted of monitoring and non-specific counselling to provide a control for time and non-specific treatment factors such as caregiver attention and expectancy. Participants were assigned to a therapist and given restricted access to the treatment platform, and were instructed to fill out a depression questionnaire on a weekly basis. Platform access allowed participants to view their depression score on the treatment platform and to message their therapist. They were informed that their assessments were to be monitored by their therapist, and were instructed to contact the therapist in the event of their symptoms deteriorating. The therapists immediately contacted participants with elevated scores. Ten control participants contacted their therapist owing to deterioration and received non-specific support while being monitored. Therapists received instructions not to use specific CBT techniques, but there was no formal procedure to check this.

### Therapists and safety measures

The therapists were four final-year clinical students who had completed clinical CBT-oriented training. Therapists received supervision (total of 8 h in a group format) from two clinical psychologists with expertise in adolescent psychopathology and delivery of iCBT treatment. Therapists reviewed chat sessions (automatically stored in the platform) for the purpose of case supervision, and study supervisors reviewed chat sessions to monitor therapists' adherence to the treatment programme.

The PHQ-9^31^ was administered to participants on weekly basis during the treatment period to monitor for depression severity (both allocations). Participants who indicated significant deterioration were immediately contacted and closely monitored, while the principal investigator and study psychiatrist evaluated eventual need to be directed to other care services. Participants were not required to inform guardians of their participation in the study, but were informed that guardian(s) were to be contacted in the event of clinical deterioration of risk. No participant had to be excluded from the study owing to deterioration.

### Outcomes

All outcome measures were administered pre- and post-treatment and after 6 months (treatment group). The primary outcome was severity of depression, measured by the BDI-II, a 21-item multiple-choice self-report instrument that measures symptoms of depression according to DSM criteria.^32^ BDI-II is intended for use from ≥13 years of age. Items are rated on a scale ranging from 0 to 3, with higher scores indicating more severe symptoms; the total score ranges from 0 to 63. Cut-off values are 0–13 (minimal), 14–19 (mild), 20–28 (moderate) and 29–63 (severe depression). The BDI-II has been found to possess excellent psychometric qualities, including high internal consistency (Cronbach's *a* = 0.91).^32^ The full MINI^27^ was administered over the phone with study therapists at baseline to assess the presence of a major depressive episode or other psychiatric disorder, and post-treatment to investigate remission from any major depressive episode. Internet-based completion of self-reported outcome measures has been shown to be a reliable method,^33^ and phone administration has been shown to be reliable in psychiatric assessment.^34^

#### Secondary outcomes

The PHQ-9^31^ was used as a complementary measure of depression symptoms. General anxiety and social anxiety were assessed using the Beck Anxiety Inventory (BAI)^35^ and the Social Interaction Anxiety Scale.^36^ The General Self-Efficacy Scale (GSE),^37^ a ten-item multiple-choice self-report scale, was used to measure optimistic self-belief and belief in personal agency to cope with demands in life. The Satisfaction With Life Scale^38^ was used to measure life satisfaction.

#### Assessment of treatment acceptance and negative experiences

The credibility scale in the Credibility Expectancy Questionnaire (CEQ)^39^ was administered after the first week of treatment to assess perceived treatment credibility. The 12-item client version of the Working Alliance Inventory^40^ was administered to iCBT participants in the third week of treatment to assess alliance. Post-treatment BDI-II scores, together with a question about negative treatment-related experiences, were used to assess potential negative effects of treatment.

### Statistical analysis

Statistical analyses were performed using SPSS version 22. Independent samples *t*-tests were used for continuous distributed variables, and Pearson's chi-squared test of independence was used for categorical data. All primary outcome measures were pre-specified in a trial registration according to the CONSORT statement.^41^ No correction for multiple comparisons was performed, as this is not current practice. Participants were included in statistical analyses according to the intention-to-treat principle. Missing data at the post-treatment and follow-up assessments were addressed using multiple imputations.^42^ Differences in primary and secondary outcomes were evaluated at post-treatment and follow-up assessments by analysis of covariance, with baseline values as covariates at the *P* < 0.05 level.^43^ Cohen's *d* effect sizes were calculated using pooled s.d. values derived from the regression model. For all measures, 95% CIs were reported as interval estimates for the true population parameters. To determine adequate sample size, power was calculated *a priori* using effect sizes that were previously reported in a meta-analysis on iCBT for anxiety and depression in youth^2^ (comparisons between guided iCBT and a wait-list control condition resulted in an overall between-group effect size of *g* = 0.72). A power analysis indicated that to achieve 80% power, with an alpha of 0.05, a sample size of 36 in each condition would be needed to detect a between-groups effect size of *d* = 0.70. To provide data on negative effects, the numbers of participants showing deterioration of 30% or more in BDI-II score from baseline to post-treatment were calculated.

## Results

The analysis comprised two components. First, it was established whether the iCBT intervention led to statistically significant improvements on measures of depression, anxiety, self-efficacy and life satisfaction. Between-group effect sizes were estimated using Cohen's *d* criteria, with effect sizes up to *d* = 0.20 considered small, *d* = 0.50 considered medium and *d* = 0.80 considered large. Second, target audience acceptance was explored by means of treatment completion, credibility and alliance measures during the course of treatment, and with open-ended questions at post-treatment assessment.

### Participant flow

**Fig. 1:**
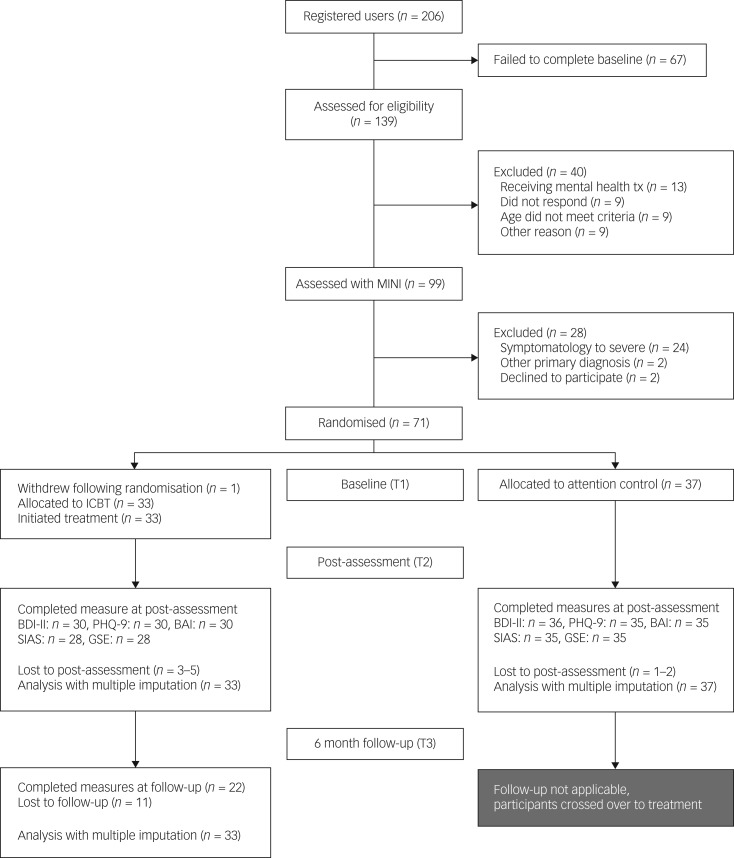
Flowchart of participants through the study.

Figure 1 presents a CONSORT diagram of participant flow through the study. During the 4-week registration period, 206 individuals registered for and 139 individuals completed the initial screening via the study website. Of those, 40 individuals were ineligible owing to current psychotherapy (13) or their age (9), because they could not be reached (9), or other reasons (9). Ninety-nine individuals were invited to the MINI assessment; of these, 28 were excluded, most often owing to severe symptomatology (24). Seventy-one individuals were eligible and were randomised. One individual declined participation following allocation to iCBT and was excluded. One individual was included in error (BDI-II score 11, not ≥14 at baseline) and was kept in the study (iCBT treatment). No differences between the conditions were detected in the baseline assessment. Analyses of male participants’ flow through the study showed that 11 of the 139 individuals that completed the baseline screening identified as male (8%). Of these, seven were excluded owing to current psychotherapy (3) or because they could not be reached (2), or because of their age (1) or severe symptomatology (1). The remaining four males were included. Table 2 describes the characteristics of the study sample. The average completion of the weekly assessment with PHQ-9 was 91% (range 81–97%). At post-treatment, primary outcome data (BDI-II) were obtained for 94% of participants (*n* = 66), and secondary outcome data were obtained for 90–93% of participants (*n* = 63–65), depending on the instrument used. Post-treatment data for MINI were obtained for 87% of participants (*n* = 61). Follow-up data at 6 months were obtained from 67% (*n* = 22) of iCBT participants. *Post hoc* analyses showed no differences between dropouts and the rest of the sample on any outcome measures at baseline.

**Table 2:** Baseline characteristics of participants

Characteristics	iCBT (*n* = 33)		Control (*n* = 37)	
	n/M	%/s.d.	n/M	%/s.d.
Female	31	93.9	35	94.6
Age	17.2	1.0	16.9	1.1
Residence				
City	10	30.3	12	32.4
Small town	14	42.4	15	40.5
Non-urban	9	27.3	10	27.0
Family				
Two-parent household	9	27.3	17	45.9
Other family constellation	24	72.7	20	54.1
Parent(s) born outside Sweden	10	30.3	8	21.6
Support modality preference				
Chat/email	27	81.8	30	81.1
Video call	1	3.0	3	8.1
No preference	5	15.2	4	10.8
Major depressive episode^a^	28	84.8	25	67.6
Depression, core symptoms	5	15.2	12	32.4
Comorbid anxiety diagnosis, any^a^	24	72.7	29	78.4
Generalised anxiety disorder	9	27.3	14	37.8
Social anxiety disorder	15	45.5	12	32.4
Panic disorder	6	18.2	8	21.6
Agoraphobia	16	48.5	15	40.5
Previous mental health contact	21	63.6	24	64.9
Previous treatment	14	42.4	13	35.1
Counsellor contact	6	18.2	5	13.5
Psychotherapy treatment	11	33.3	7	18.9
Psychotropic medication	3	9.1	1	2.7
Current treatment	5	15.2	10	27.0
Counsellor contact	4	12.1	5	13.5
Psychotropic medication	1	3.0	5	13.5

a. Confirmed by the Mini-International Neuropsychiatric Interview.

### Outcome measures

**Fig. 2:**
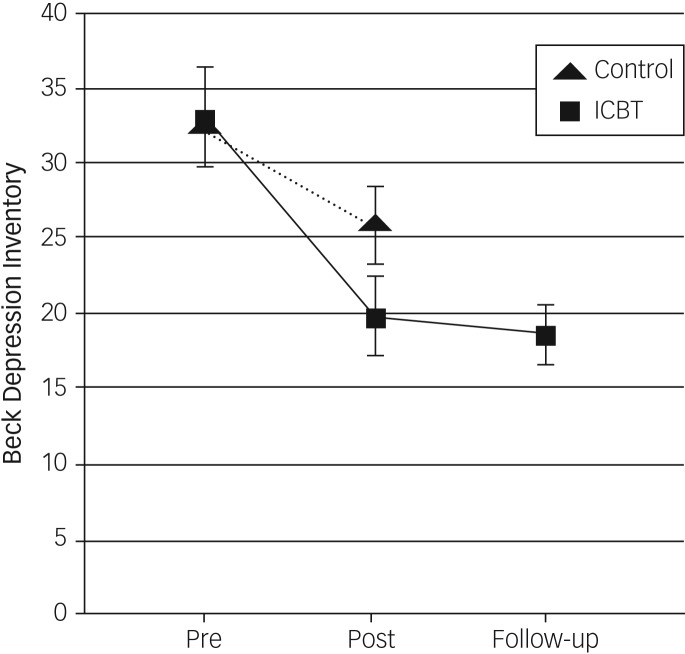
Change over time in depression severity (95% CIs).

Table 3 presents pre- and post-treatment assessments including effect sizes, means and s.d. for both groups, together with the follow-up assessment for the treatment group. Analyses with ANCOVA for BDI-II difference at post-treatment assessment, with covarying baseline scores, revealed a significant effect between the treatment group and the control group (*F* = 6.18, d.f. = 1,67, *P* < 0.05). The corresponding between-groups effect size was moderate, near large (Cohen's *d* = 0.71, 95% CI 0.22–1.19). Figure 2 illustrates within-group improvements on the BDI-II. The ANCOVA for post-treatment change in self-efficacy (GSE) revealed a significant effect between the iCBT condition and the control condition (*F* = 11.82, d.f. = 1,67, *P* < 0.001.). The corresponding effect size between groups was large (Cohen's *d* = 1.33, 95% CI = 0.80–1.85). The ANCOVAs for post-treatment change, with covarying baseline scores, showed no significant effect between groups for the PHQ-9 (*F* = 1.03, d.f. = 1,67, *P* = 0.313), the BAI (*F* = 0.02, d.f. = 1,67, *P* = 0.881), the Social Interaction Anxiety Scale (*F* = 0.90, d.f. = 1,67, *P* = 0.347) or the Satisfaction With Life Scale (*F* = 0.864, d.f. = 1,67, *P* = 0.365).

**Table 3:** Means, s.d. and effect sizes (Cohen's *d*) with 95% CIs for continuous outcome variables

Measure	Mean (s.d.)			Effect size, Cohen's *d* (95% CI)		
	Pre-treatment	Post-treatment^a^	6-month^a^	Between-group pre to post	Within-group, pre to post	Within-group, pre to 6 months
BDI-II						
iCBT	33.1 (9.4)	19.9 (7.2)	18.6 (1.8)	0.71 (0.22–1.19)*	1.22 (0.76–1.67)***	1.08 (0.64–1.50)***
Control	32.3 (10.2)	25.2 (7.8)			0.75 (0.38–1.11)***	
GSE						
iCBT	19.8 (4.7)	24.4 (2.8)	23.6 (2.8)	1.33 (0.80–1.85)***	0.94 (0.52–1.35)***	0.50 (0.14–0.86)**
Control	19.0 (5.5)	20.4 (3.3)			0.29 (−0.41–0.62)	
PHQ-9						
iCBT	15.2 (4.8)	9.7 (2.9)	10.2 (1.5)	0.36 (−0.10–0.84)	0.97 (0.55–1.38)***	0.74 (0.35–1.11)***
Control	14.9 (5.0)	10.8 (3.0)			0.76 (0.39–1.13)***	
BAI						
iCBT	27.0 (12.1)	20.6 (9.0)	17.3 (4.5)	0.14 (−0.33–0.60)	0.70 (0.31–1.07)***	0.75 (0.35–1.13)***
Control	25.0 (11.6)	19.4 (8.6)			0.66 (0.30–1.01)***	
SIAS						
iCBT	45.6 (16.0)	39.3 (13.8)	41.7 (12.1)	0.16 (−0.30–0.63)	0.62 (0.25–0.99)**	0.19 (−0.15–0.54)
Control	45.1 (13.7)	41.4 (11.8)			0.29 (−0.37–0.62)	
SWLS						
iCBT	12.3 (5.0)	14.2 (2.6)	15.3 (2.5)	0.11 (−36–0.58)	0.33 (−0.15–0.69)	0.46 (0.10–0.82)*
Control	13.8 (5.7)	13.9 (3.0)			0.38 (−0.05–0.60)	

a. Model-derived means differences (s.d.).

Intention-to-treat analysis adjusted for baseline score. BDI-II, Beck depression Inventory; GSE, General self-efficacy scale; PHQ-9, Patient health Questionnaire: BAI, Beck Anxiety Inventory; SIAS, Social Interaction Anxiety Scale; SWLS, Satisfaction With Life Scale.

*P < 0.05. **P < 0.01. ***P < 0.001.

### Response and remission rates

Table 4 presents descriptive statistics of participants' improvement from baseline to post-treatment. Response was defined as a 30% or more decrease in symptoms on the BDI-II from baseline to post-treatment. When missing cases were categorised not to have changed, a higher proportion of participants in the iCBT group than control participants responded to treatment (χ^2^(1), *P* < 0.05). Additionally, any decrease of 50% or more in BDI-II score from baseline to post-treatment was investigated, and a significant difference between groups was found (χ^2^(1), *P* < 0.01). With missing cases categorised not to have changed, a significantly higher proportion of iCBT participants than control participants no longer met DSM-IV criteria for a major depressive episode at post-treatment assessment (χ^2^(1) = 16.37, *P* < 0.001).

**Table 4:** Response and remission, based on scores on the main outcome measure Beck Depression Inventory II, and DSM-IV criteria for major depressive episode^a^

Measure, *n* (%)	iCBT (*n* = 33)^b^	Control (*n* = 37)^b^
≥30% decrease in score from baseline to post assessment	20 (60.6)*	12 (32.4)
≥50% decrease in score from baseline to post assessment	14 (42.4)**	5 (13.5)
No longer meet DSM-IV criteria for major depressive episode^c^	20 (71.4)***	4 (16.0)

a. Confirmed by the Mini-International Neuropsychiatric Interview.

b. Missing cases (iCBT *n* = 3, Control *n* = 1) considered not to have changed.

c. Baseline sample *n* = 53. Missing cases (iCBT *n* = 5, Control *n* = 4) considered not to have changed.

*P < 0.05, **P < 0.01, ***P < 0.001.

### Negative effects

Post-treatment BDI-II scores and an open-ended question about negative treatment-related experiences were used to assess negative effects associated with treatment. Based on completers, one participant in the iCBT group (3%) and three participants in the control group (8%) showed a deterioration of 30% or more in BDI-II score post-treatment. With missing cases categorised to have deteriorated significantly, the rate in the iCBT group was 12.1% (*n* = 4). When probed for experienced negative effects in the open-ended question post-treatment, five iCBT participants reported occasional stress due to tempo and workload in treatment, or at times feeling worse while processing treatment content.

### Programme use and treatment acceptability

Participants completed an average of 6.24 chat sessions of the eight available (s.d. = 2.60) and 6.48 modules of the eight available (s.d. = 2.43). Of these participants, 70% completed the full treatment, defined as completing in total eight modules and sessions, and 9.1% of participants completed less than 50% of treatment. One participant in the control group did not complete all weekly assessments or the post-treatment assessment, and was considered to have dropped out. Spearman correlations revealed no dose-response effect from treatment. Table 5 presents an overview of treatment completion. Participants logged on to the study website 40.2 times on average (range 6–67, s.d. = 16.1), which corresponds to five times per week. Excluding written homework completion and chat sessions, participants sent 10.6 messages (range 2–23, s.d. = 6.2) to their therapist. Therapists spent an average of 47.3 min (s.d. = 5.7) on each participant every week, and sent 30.4 messages (range 8–53, s.d. = 9.3) to each participant, excluding chat sessions. iCBT participants' ratings of treatment credibility (CEQ scale) showed an average rating of M = 22.07 (s.d. = 2.23) out of a maximum total of 27 (highest credibility score). The average score on Working Alliance Inventory items was 5.61 (s.d. = 0.91) out of a maximum of 7 (highest satisfaction).

**Table 5:** Number of participants in the iCBT group completing each module and chat session

	Number of completed modules and sessions							
	≥1	≥2	≥3	≥4	≥5	≥6	≥7	≥8
Module, *n* (%)	33 (100)	29 (87.9)	28 (84.8)	27 (81.8)	26 (78.8)	25 (75.8)	22 (66.7)	18 (54.5)
Chat session, *n* (%)	33 (100)	30 (90.1)	29 (87.9)	28 (84.8)	26 (78.8)	25 (75.8)	22 (66.7)	12^a^ (36.4)

a. Four participants completed nine chat sessions.

### Intervention development and cost

The chat function was developed partly using open source code and was integrated into the existing treatment platform over a 3-week period, including tests, feedback and optimisation. The total cost for web development and service during the treatment period was estimated to be €3800 (based on the average salary for a software and system developer in the public sector), which equals to €54 per participant. We added €23 for each hour of treatment, which is the hourly salary for a therapist in training, including tax. Therapists on average spent 47.3 min per participant each week, which was calculated as 60 min. Based on estimates for a digital primary care provider service in Sweden,^44^ an indirect cost per consultation of €24 (management, office space, administration and support) was added for each therapist hour. Based on these numbers, the total intervention cost per participant amounted to approximately €430 (€54 web costs + €376 for 8 therapist hours). The actual costs were low, as therapists or supervising clinicians were not paid, and we did not collect data to allow for a health economic evaluation.

## Discussion

The aim of the present study was to investigate the effects of a therapist-guided internet-supported CBT treatment programme for adolescent depression, delivered in the form of weekly chat sessions and online modules. We hypothesised that the inclusion of real-time therapist support would positively affect clinical outcome and participant engagement, compared with iCBT programmes with limited or no therapist input. The study confirmed that the intervention, in comparison with attention control, resulted in a significant decrease in depressive symptoms on the primary outcome (*d* = 0.71), as well as a large effect on self-efficacy (*d* = 1.33), and that treatment effects for the treatment group seemed to be maintained over 6 months. Furthermore, the intervention seemed to facilitate clinical recovery, as it led to partial remission from a major depressive episode (DSM-IV criteria) in three of four participants in the iCBT group. Although direct comparison was difficult owing to differences in population, study setting and assessment, the effect size found in our study was comparable with overall pooled effects (*g* = 0.76) for iCBT programmes targeting youth depression that have been compared with non-active control conditions,^2^ and somewhat smaller than the effect size reported (*d* = 0.94) in the one controlled study that previously evaluated CBT in the form of (group) chat sessions against waitlist.^14^ Compared with face-to-face CBT programmes evaluated against an active control group,^45^ our outcomes were favourable. Another positive finding was that only one participant (12.1% with missing cases categorised as having deteriorated) showed significant deterioration following iCBT treatment. By comparison, it has previously been reported that about 14–24% of children and adolescents experience an elevation in distress during the course of mental health interventions.^46^ A study that evaluated iCBT in treatment of adult depression in primary care settings reported a 10% deterioration following treatment.^47^ The intervention did not demonstrate a significant reduction in anxiety symptoms, despite 1 week of treatment addressing anxiety. It is possible that the treatment content was insufficient, and, since the content was delivered rather late in treatment, not all participants received it. However, moderate improvements in anxiety were shown in both allocations post-treatment. It may be that the effect relates to the assessment and therapist interaction during the screening process and treatment period.

Specifically, the inclusion of therapist sessions may have played a part in the positive findings for depression symptoms and treatment engagement. Previously, preventive and self-help iCBT programmes for young people have tended to experience high attrition and/or limited clinical effect,^9^^,^^10^^,^^15^ with indications that therapist-guided iCBT and approaches including face-to-face contact may improve outcomes.^14^^,^^15^ The current intervention is an example of a ‘blended’ treatment approach, emphasising active therapist collaboration in combination with online self-help material.^16^^,^^21^ Chat communication is favoured among young people,^11^^,^^22^ and benefits associated with eliminating the pressure of sitting opposite a physical person have previously been reported for chat-based helplines,^22^ indicating the potential of the chat medium to reduce stigma. Similar advantages were found in the present study. Participants showed a high level of self-disclosure early in treatment, and rated the treatment as credible and their therapist working alliance as positive. The attrition rate being relatively low, and not in line with the high attrition rates sometimes seen for internet interventions, is a complementary and promising result with regards to treatment acceptability.

The study sample included predominantly girls. Similar gender distributions have consistently been reported for online and chat-based help lines and counselling services for young people.^14^^,^^22^^,^^48^^,^^49^ A study investigating the gender distribution among adolescents and young adults accessing in-person *v.* web-based counselling centres found that adolescent males were more likely to access physical centres than online centres.^49^ The authors discussed that the gender distribution in online settings may relate to the fact that males are more likely than females to be influenced by others, e.g. by family, to attend mental health services, and that this in turn may act against young men's uptake of online services, given that these are dependent on self-motivation. There are aspects of the online setting that may appeal more to females than males, and the underlying causes should be further explored and addressed in future studies; for example, it could be investigated whether more targeted outreach to adolescent males could increase uptake.^49^

### Strengths and limitations

The present study addresses a gap in the literature concerning the efficacy and feasibility of mental health interventions that include real-time chat support. The study design, use of reliable and empirically sound primary outcome measures, semi-structured diagnostic interview, and sufficient power to detect differences between groups are strengths of the current study. Limitations include the fact that the study design did not allow for comparison between groups at follow-up, and that there was data loss at follow-up in the iCBT group. *Post hoc* tests showed no difference between completers and non-completers in terms of depression severity post-treatment, and missing data were addressed using multiple imputations. Still, there was some risk of overestimation of the results. We evaluated the treatment against an attention control; as participants in the control group had less therapist contact, it cannot be ruled out that part of the obtained effect was associated with general caregiver attention. Weaknesses relating to assessment include the post-treatment clinician-administered interviews not being blinded, which may have affected the results. In addition, learning effects may have interfered with the results for the secondary outcome (PHQ-9), as the instrument was used for weekly assessments, and the sample size did not allow reliable investigation of predictors. Finally, the gender distribution limited the generalisability of the findings.

### Conclusion

This study demonstrates that a combination of clinically meaningful effects, target audience acceptability and real-world feasibility can be achieved with iCBT for the treatment of adolescent depression. The findings on outcome further indicate that therapist support plays a part in improving clinical outcome in treatment of young populations. The findings on treatment completion and alliance indicate that therapist contact in the form of chat communication can produce a stable and positive therapeutic relationship, and provide a meaningful treatment for young individuals. Further research is warranted to verify results on clinical effects, including the role of therapist support for the long-term clinical- and cost-effectiveness of iCBT treatment.

### Clinical implications

A common criticism of controlled studies is that study populations are too restricted to reflect the true clinical population. For the adolescent population, however, it can be questioned whether the clinical population can be properly approximated, given that it is estimated that only 20% of young people with mental healthcare needs receive treatment, and that social stigma is a significant barrier for many to seek treatment. This study has helped to reveal the population to be expected when providing an alternative and direct pathway to care, with terms and treatment designed to improve young individuals’ autonomy (parental consent was not required) and reduce stigma. The intervention appealed to a broad range of young people, including individuals with previous mental healthcare history, as well as those who had not presented for standard care or school counselling services (36%). The depression severity and comorbid psychopathology found were noteworthy, as was the high proportion of girls. The findings indicate that internet-based treatment approaches are needed, and that adjustments to the conditions under which young people access mental health services have the potential to make intervention feasible for many more young individuals. Results for guided internet intervention and digital primary care models are promising in terms of cost-effectiveness.^44^^,^^50^ Multiple concurrent sessions (increasing capacity) is common practice in chat-based helplines for young people.^22^ The next step is to investigate whether this can be generalised to chat sessions in iCBT. If so, the cost savings in comparison to standard treatment delivery could be substantial. The present study makes a promising contribution to the development of (internet) interventions that overcome structural and individual barriers, and appropriately address the needs of young people in need of mental healthcare assistance.

## Supplementary material

For supplementary material accompanying this paper visit http://dx.doi.org/10.1192/bjo.2018.18. click here to view supplementary material
