# Supplementary material for: Chat- and internet-based cognitive–behavioural therapy in treatment of adolescent depression: randomised controlled trial
Source: BJPsych Open. 2018 Jun 26;4(4):199–207. doi: 10.1192/bjo.2018.18 (PMC6034465; doi:10.1192/bjo.2018.18)
Supplement: Supplementary file 1 [file S2056472418000182sup001.pdf]

Appendix A. Screenshots of the iCBT treatment, Topooco et al., 2018

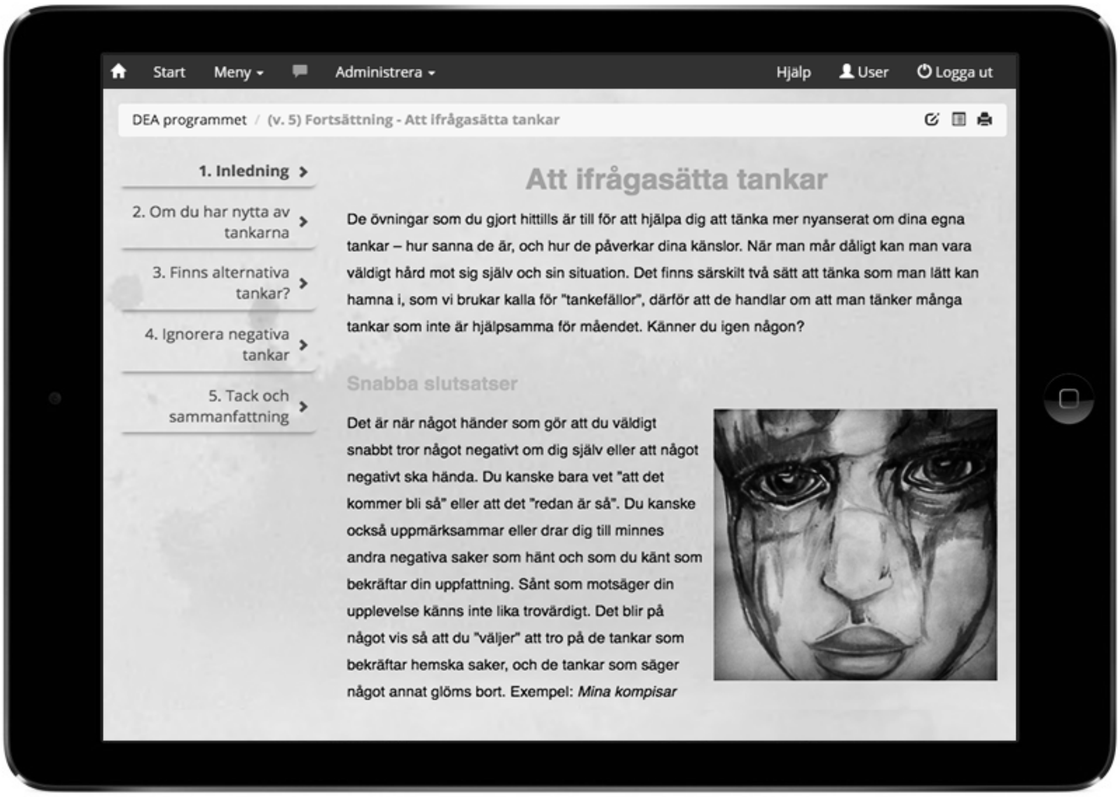

1. Treatment module

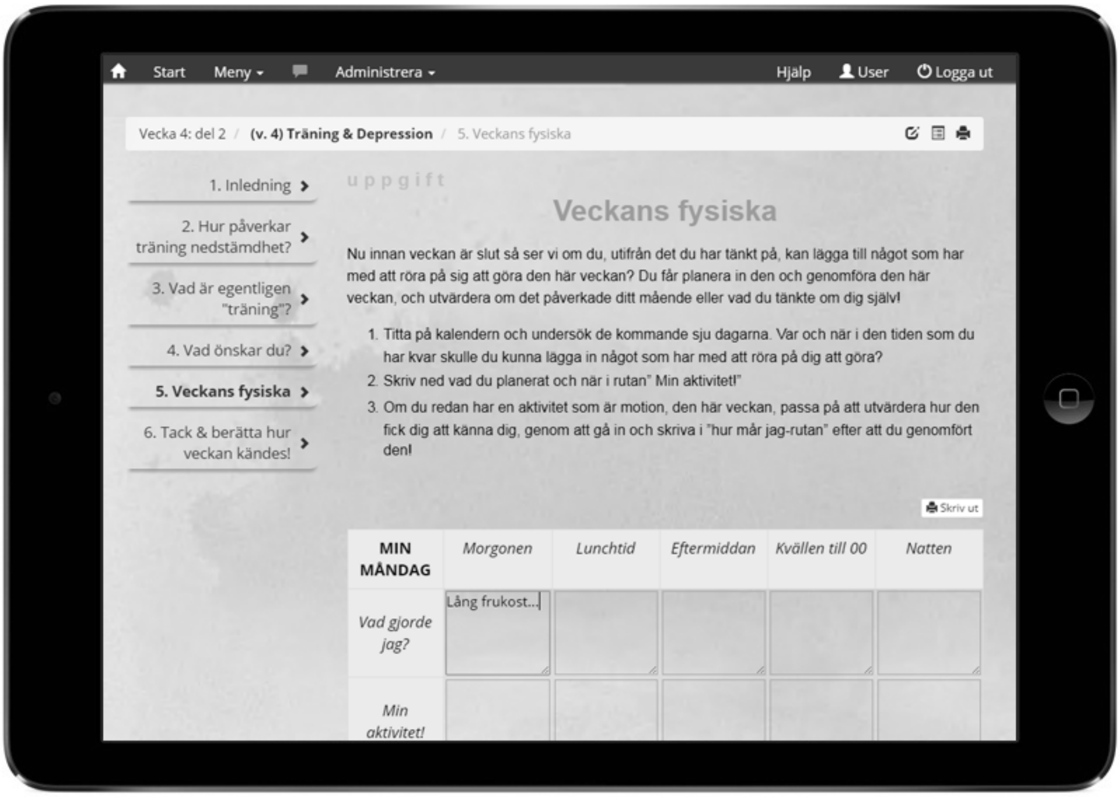

2. Treatment module, homework assignment

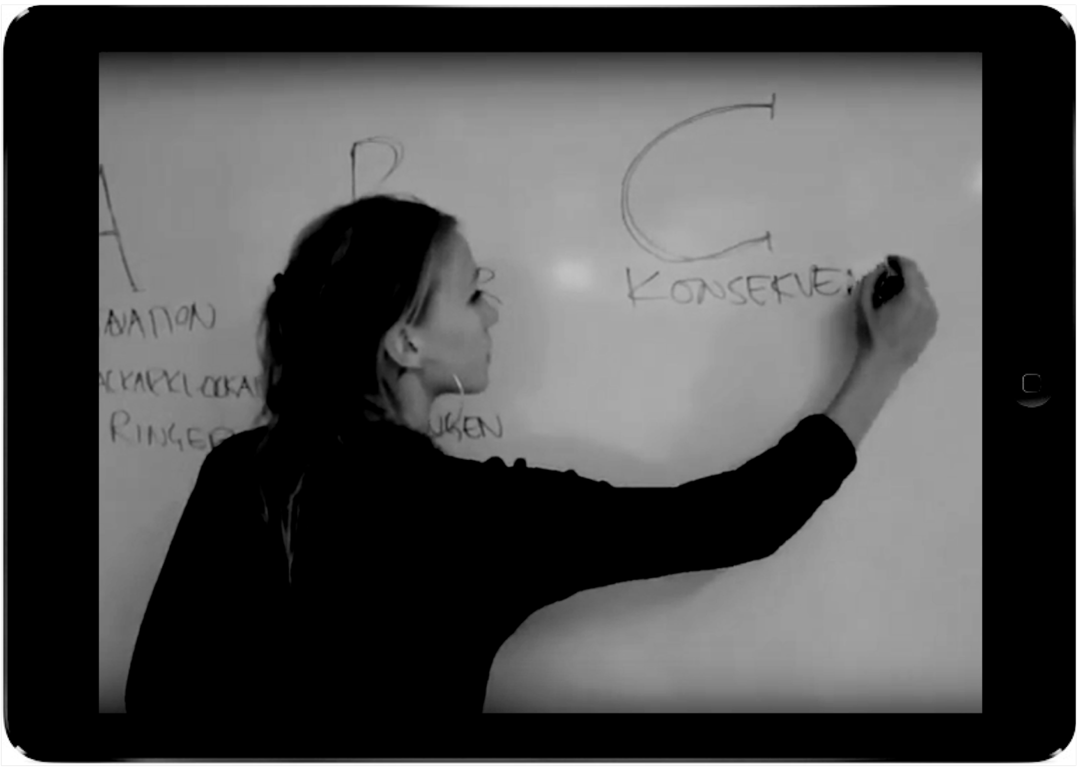

3. Treatment module, movie-clip

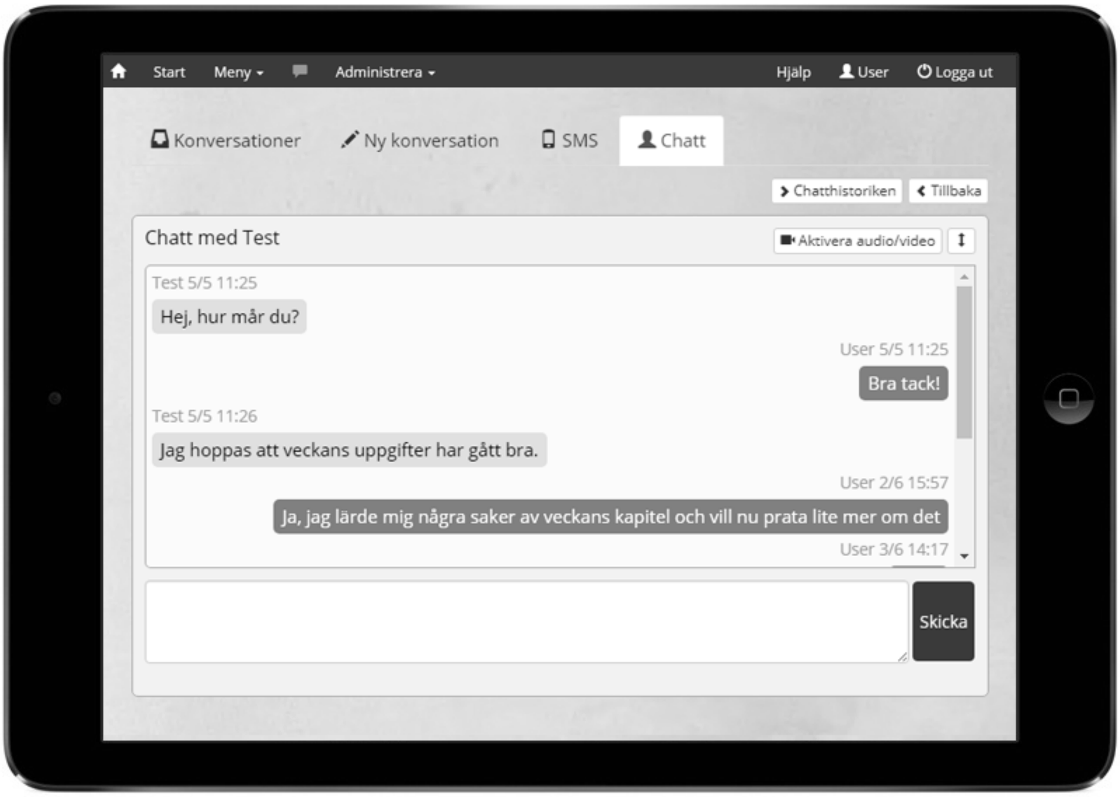

4. Chat session interface

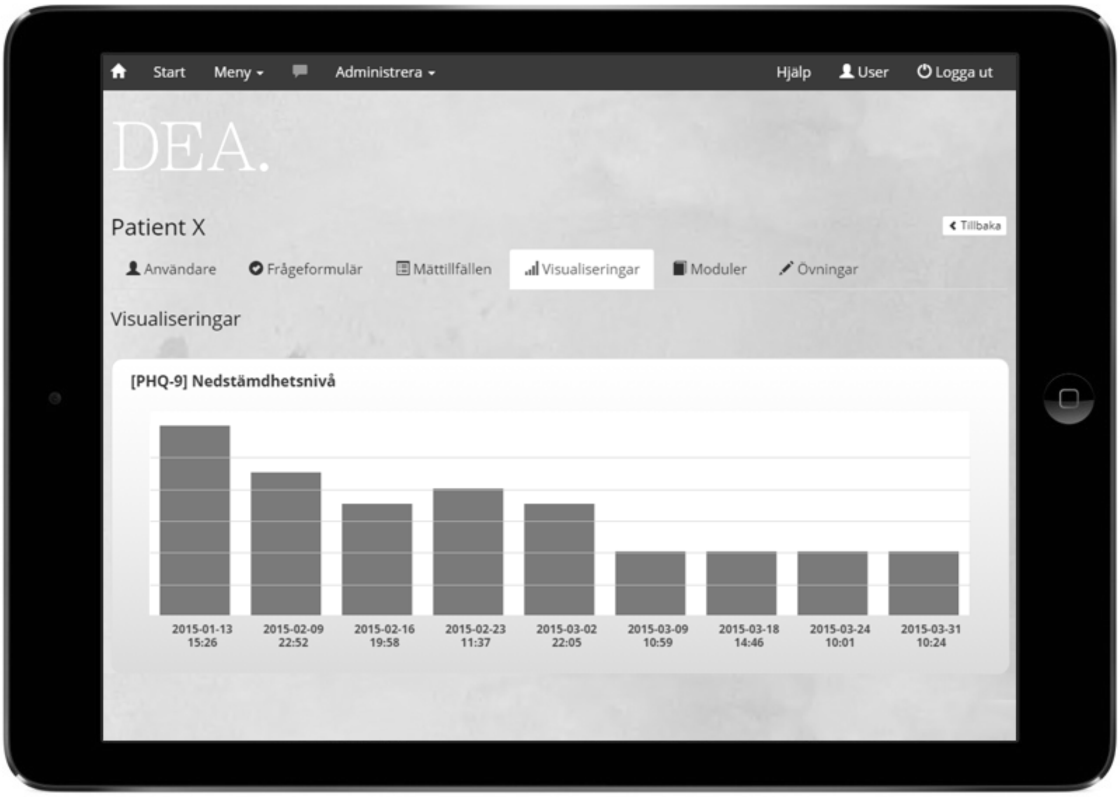

5. Weekly assessment overview (participant/therapist view)
